# Is advanced bipolar vessel sealing device really effective in decreasing the pelvic lymphocele ratio in open surgery for endometrial cancer?

**DOI:** 10.55730/1300-0144.5559

**Published:** 2023-02-01

**Authors:** Mehmet Ali NARİN, Sevda BAŞ, Sevtap SEYFETTİNOĞLU, Tuba YAR, Raziye NARİN

**Affiliations:** Department of Obstetrics and Gynecology, University of Health Sciences, Adana City Education and Research Hospital, Adana, Turkey

**Keywords:** Endometrial cancer, lymphadenectomy, pelvic lymphocele, advanced bipolar sealing device

## Abstract

**Background/aim:**

We aim to show pelvic lymphocele (PL) rates in patients who were operated for endometrial cancer (EC) and underwent systematic paraaortic bilateral pelvic lymph node dissection (PABPLND) with advanced bipolar vessel sealing device (ABVSD)

**Materials and methods:**

The medical files of all patients who underwent open surgery for EC between January 2017 and December 2021 were retrospectively analyzed. One hundred three patients who operated with the diagnosis of high-intermediate and high-risk endometrial cancer were included. Systematic PABPLND was performed with total abdominal hysterectomy with or without bilateral salpingo-oophorectomy during surgery to all patients. All operations were performed by same three surgeons who were expert in their field. While the lymph packages were removed during surgical dissection, the distal afferent and proximal efferent lymphatic channels were sealed with LigaSure™ blunt tip sealer/divider (Medtronic, Covidien, USA). The patients were scanned with computed tomography (CT) between 8 and 12 weeks postoperatively. Lymphocele diagnosis was confirmed by radiologists and largest diameter was recorded. Clinical-pathological findings of all patients were recorded.

**Results:**

Mean age and body mass index (BMI) of all participants were 58.6 ±10.2 years and 28.1± 5.6 kg/m^2^. The most histopathological findings were endometrioid type (84.5%) and grade 2 (44.2%) ECs. The pelvic lymphocele (PL) was detected with CT in 24 of 103 patients at 8 to 12 weeks postoperatively. Only two PL patients were symptomatic. The first patient had symptoms of pelvic fullness and compression while the second patient had infected image. PL was located to right pelvic area in first case while the second was located on the vaginal cuff.

**Conclusion:**

The dissection and sealing of major lymph vessels were achieved during the removal of all lymph packages with LigaSure™ blunt tip laparoscopic sealer/divider. The use of advanced bipolar systems can reduce the formation of PL in lymph node dissection in endometrial cancer.

## 1. Introduction

Endometrial cancer (EC) is the most common malignancy of the female genital tract [1]. Systematic paraaortic and bilateral pelvic lymphadenectomy (PABPLND) is still a prominent step in the surgical staging and treatment of several gynecologic cancers, including EC [2].

Pelvic lymphocele (PL) is the most common and problematic postoperative complication of lymphadenectomy. It is defined as the collection of lymphatic fluid without discrete epithelium, which results from the transection of afferent lymphatic vessels during surgical dissection and is frequently located around the iliac vessels. Although the pathophysiology of lymphocele formation is not fully understood, the number of removed lymph nodes, the lack of ligation of the lymphatic vessel, extensive lymphadenectomy, radical surgery, perioperative radiation, presence of lymph node metastasis, and high body mass index (BMI) are considered responsible factors. The incidence of PL varies between 1% and 49% [3–10]. They are usually discovered incidentally and have no clinical significance but sometimes show serious symptoms mainly related to their significant size. As a result, adjuvant treatment is delayed due to PL, and significant problems may occur [11,12]. Many techniques, such as nonclosure of pelvic peritoneum, absence of retroperitoneal drainage, omentoplasty, or fibrin application, have been described to decrease the incidence of PL after open lymphadenectomy [11]. However, the effect of the described procedures on reducing the incidence of PL is not very evident

LigaSure™ blunt tip sealer/divider (Medtronic/Covidien, USA) is an advanced bipolar vessel sealing device (ABVSD) primarily used in laparoscopic surgery and designed to provide combination of pressure and energy to create a consistent seal with each application. In our hospital, we have used this device since 2015 in both open and laparoscopic gyneco-oncological surgery. Considering the highest burst pressure and fastest sealing time, we think their use will be appropriate, especially in decreasing the incidence of postoperative PL by firmly sealing the lymphatic vessels [13]. To our knowledge, there is no up-to-date study, and it is the first study to show whether using ABVSD for lymphadenectomy will reduce the occurrence of PL in endometrial cancers that underwent open lymphadenectomy.

## 2. Materials and methods

The medical files of all patients who underwent open surgery for EC between January 2017 and December 2021 were retrospectively analyzed in the hospital’s electronic information system, and retrospective analysis was performed. A total of 103 patients who were operated on with the diagnosis of high-intermediate and high-risk EC by using the ABVSD were included in the study at the Department of Gynecologic Oncology of the University of Health Sciences Adana City Education and Research Hospital. Detailed informed consent for surgery was obtained from all patients. Systematic PABPLND was performed with total abdominal hysterectomy with or without bilateral salpingo-oophorectomy during surgery for all patients. Approval from the University of Health Sciences institutional review board was obtained (Decision no: 114/2211). Patients who underwent radical hysterectomy or had synchronous primary tumors at the time of surgery, patients with uterine sarcoma, hematologic or coagulation disorders, previous thromboembolic or lymphatic system disease and radio-chemotherapy were excluded from the study.

**Figure 1:**
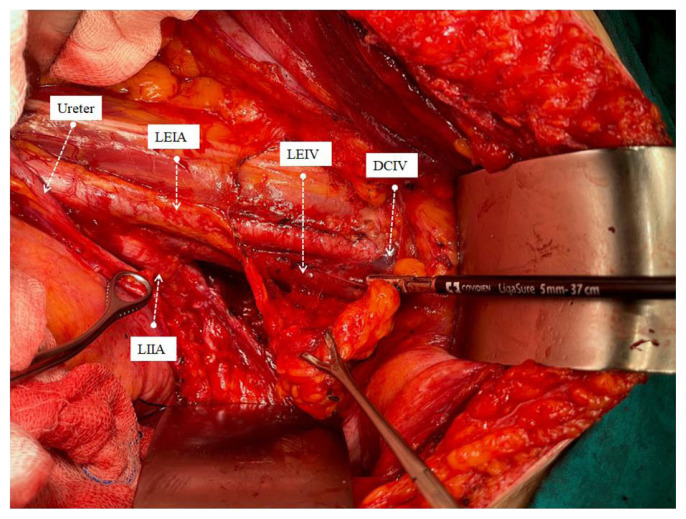
Removal of left iliac lymph package with the sealing of afferent distal lymph vessels. ***♀***LEIA: Left external iliac artery, LEIV: Left external iliac vein, DCIV: Distal circumflex iliac vein, LILA: Left internal iliac artery.

**Figure 2:**
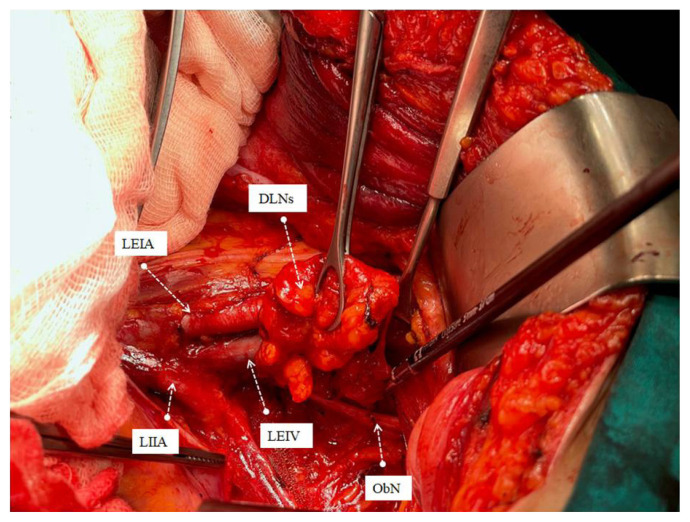
Partial removal of left obturator lymph package with the sealing of afferent distal lymph vessels. ***♀***LEIA: Left external iliac artery, LEIV: Left external iliac vein, Ob N: Obturator nerve, LILA: Left internal iliac artery, DLN: Dissected lymph nodes.

**Figure 3:**
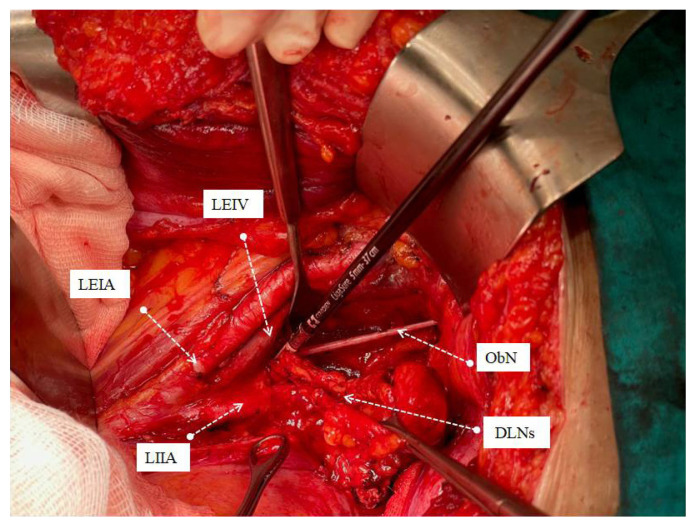
Total removal of left obturator lymph package with the sealing of efferent proximal lymph vessels. ***♀***LEIA: Left external iliac artery, LEIV: Left external iliac vein, Ob N: Obturator nerve, LILA: Left internal iliac artery, DLN: Dissected lymph nodes.

**Figure 4:**
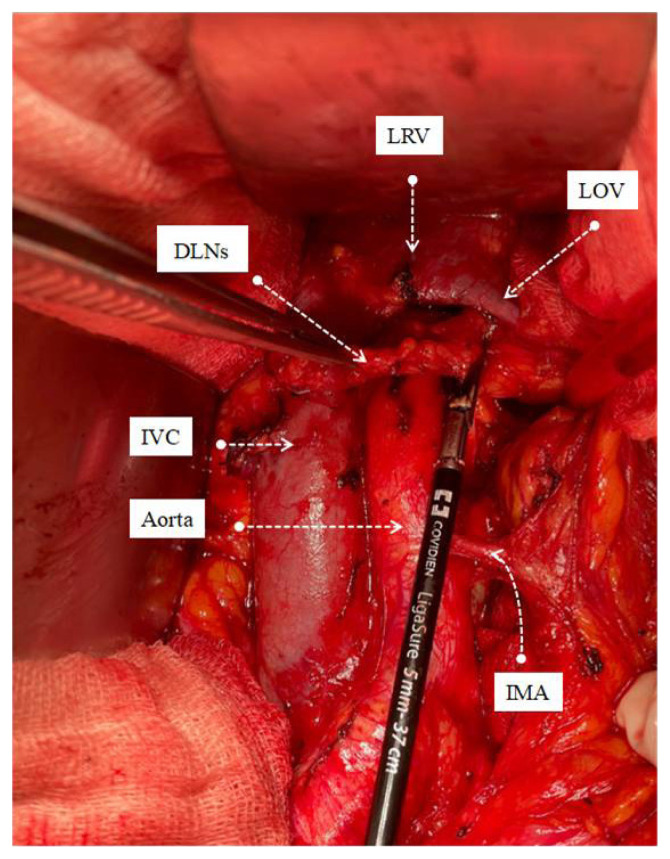
Removal of left paraaortic lymp package with the sealing of efferent proximal lymph vessels. ***♀***IVC: Inferior vena cava, LRV: Left renal vein, LOV: Left ovarian vein, IMA: Inferior mesenteric artery, DLN: Dissected lymph nodes.

All operations were performed by the same three surgeons who were experts in their field after 3 years of gynecological oncology fellowship training program (M.A.N., S.B., S.S.). Systematic PABPLND is defined as removing all lymphatic tissue caudally from the bilateral deep circumflex iliac veins and cranially up to both renal veins, including paracaval, paraaortic, interaortacaval, common iliac, external iliac, internal iliac, and obturator lymph nodes. While the lymph packages were removed during surgical dissection, the distal afferent and proximal efferent lymphatic channels were sealed with LigaSure™ blunt tip sealer/divider (Medtronic, Covidien, USA) (Figures 1–4). A surgical clip has never been used for lymphatic channel sealing. Retroperitoneum was not sutured in any operation, and the vaginal cuff was closed in all cases. Once the surgery was completed, a 10-mm Jackson-Pratt drain was mostly placed in the douglas space with the option of operator choice and removed when the amount of fluid drained was less than 100 mL per day since no bleeding, or serous fluid was noticed among our patients [14]. The patient’s well-being was evaluated by daily clinical examination after the operation. They received low molecular weight subcutaneous heparin therapy at least 4 weeks after the operation.

All patients were scanned with computed tomography (CT) between 8 and 12 weeks postoperatively. Radiologists confirmed lymphocele diagnosis, and the largest diameter was recorded. Symptomatic patients were followed more frequently with ultrasonography or CT. All patients were also controlled for PL and recurrent disease with CT 6 months after the operation, as CT scan is performed on all patients in our clinic in the 2nd/3rd and 6th months. We compared the clinicopathological characteristics of the patients who developed PL or not after performing PABPLND with ABVSD.

### 2.1. Statistical analyses

SPSS 18.0 (IBM, Chicago, USA) program was used for statistical analysis. The distribution normality of continuous variables was checked with the Kolmogorov–Smirnov/Shapiro–Wilk tests. In descriptive statistics, numbers and percentages were given for categorical variables, while mean and standard deviation were given for numerical variables. Chi-square and Fisher’s exact tests were used for categorical variables where applicable. Comparisons of numerical variables between two groups were made with Student’s *t*-test in cases where normal distribution was provided and with the Mann–Whitney U test in cases where it was not. The significance level was accepted as p < 0.05 in the evaluation.

## 3. Results

The patient’s demographic and clinical characteristics are shown in Table 1. The mean age and BMI of all participants were 58.6 ±10.2 years and 28.1± 5.6 kg/m2. There were 18 patients with a previous history of abdominal surgery, and the mean operative time was 228.0 ± 63 min, while drains were placed in 82 patients (79.6%). According to the International Federation of Gynecology and Obstetrics (FIGO) 2009 staging system, the majority of the participants were stage I (N = 69, 67%), while those who received chemotherapy and radiotherapy after the operation were 43 (41.7%) and 57 (55.3%) participants, respectively.

The participants were divided into two groups according to the presence of PL. Systematic paraaortic lymphadenectomy was performed in all our cases. We observed that as the total number of removed lymph nodes, paraaortic lymph nodes, and metastatic lymph nodes increased, the presence of PL increased significantly (p = 0.005, 0.008, 0.024, respectively).

Histopathological findings of the tumors are presented in Table 2. Most patients had endometrioid type (84.5%) and grade 2 (44.2%) ECs. Tumor diameter was 4 cm or more in 61 (59.2%) cases, and lymphovascular space invasion (LVSI) was observed in 32 (31.1%) cases. Tumor localization was mostly related to upper segment of the uterus (73.8%). The median of total lymph nodes dissected in operation was 36 (9–133), and the metastatic was 2 (1–27).

Lymphocele was detected with CT in 24 of 103 patients at 8 to 12 weeks postoperatively. The incidence of PL was determined as 23.3% in these cases. The mean largest diameter of the lymphocele was 4.4 ± 2.0 cm. The incidence of symptomatic lymphocele was 1.9% (2/103), while asymptomatic lymphocele was 21.3% (22/103).

No statistical comparison was made in terms of PL symptoms because only two patients were symptomatic. The first patient had a 9.4 cm in diameter lymphocele and symptoms of pelvic fullness and compression. In the second patient, the lymphocele diameter was 4.4 cm, and the infected image was dominant. The first case was followed up with interventional pigtail while the second was followed up with antibiotic therapy, and they were able to receive their adjuvant treatment without interruption. No lymphocele was detected in any of the patients, including the symptomatic 2 cases at the 6th month, which was confirmed with CT.

All variables, including BMI, tumor histology, grade, diameter, localization, LVSI, stage, adjuvant therapy, drain use, previous abdominal surgery, the total number of lymph nodes removed, and the number of metastatic lymph nodes, which are thought to be taking part in developing lymphocele formation, were recorded. A statistical comparison of the groups with and without lymphocele is shown in Table 3. There was a significant difference in BMI, histology, grade, LVSI, stage, adjuvant therapy, previous abdominal surgery, and total extracted and metastatic LN (p < 0.05). On the other hand, there was no significant difference in tumor diameter, localization, and drain usage.

## 4. Discussion

We examined the postoperative lymphocele development rates in patients who were operated on for endometrial cancer and underwent systematic PABPLND with LigaSure™ blunt tip laparoscopic sealer/divider.

In the literature, the rate of postoperative lymphocele is given in a wide range, such as 1 to 59 percent, and it is stated that 5 to 18 percent of these can be symptomatic [15–17]. Most of the PL is asymptomatic and incidental without clinical significance. However, enlarged lymphoceles compress the surrounding tissues and may cause symptoms such as pelvic pain, leg edema, hydronephrosis, venous thrombosis, and infection. Symptomatic lymphoceles negatively affect the quality of life and may cause sepsis secondary to abscess formation due to chemotherapy-induced bone marrow suppression in cases requiring adjuvant chemotherapy. Although the total symptomatic lymphocele rate was reported as 1.9 percent in both laparoscopic and laparotomic surgeries using different surgical techniques, the risk was reported to be higher in open surgery in the most recent metaanalysis conducted in 2022 [11]. In our study, the rate of symptomatic PL was quite low compared to endometrial cancers performed by laparotomy (1.9%). This difference may arise due to the relatively low number in our study group.

Extensive lymphadenectomy, the total number of lymph nodes removed, deficiencies in ligation of lymphatic vessels, presence of metastatic lymph nodes, postoperative radiotherapy, and presence of drain and prophylactic anticoagulant use are thought to be responsible for PL formation [18–20]. However, the net contribution of these factors has yet to be demonstrated in prospectively designed studies. We also presented the variables that affect the formation of PL in Table 3.

Unlike most of the studies in the literature, our primary aim was to show whether the use of LigaSure™ blunt tip laparoscopic sealer/divider would be effective in open surgery and, secondarily, the effect of systematic paraaortic lymphadenectomy on PL rates. Almost all of the studies in the literature related to PL rates either did not involve paraaortic lymphadenectomy or were performed in the form of sampling [21,22]. The median of total lymph nodes was 36 (9–133) and the mean of paraaortic lymph nodes was 14.4 ± 10.9, so removed total lymph node numbers during the operation were higher than the number given for systematic lymphadenectomy in the literature. Still, the rate of symptomatic lymphocele is very low, it did not have a significant effect on symptomatic PL formation, and this situation is probably related to the surgical technique [20].

Significant advances have been made in advanced bipolar technology in recent years. LigaSure systems are one of the essential devices used in this sense. LigaSure has been used in our hospital since 2015, and since the central government purchased the device, it has been the only bipolar energy modality that could be used until 2022. We have been using the LigaSure™ blunt tip laparoscopic sealer/divider (37 cm) for lymphadenectomy since 2016. We wanted to show the effect of systematic open PABPLND performed with LigaSure™ on PL formation in endometrial cancers. In the study of Tsuda et al. involving 321 gynecological cancer cases, an 18.8 cm long short-tipped LigaSure was used, and it was emphasized that it reduced lymphocele rates compared to conventional bipolar systems [20]. Our device is 37 cm long and has a wider tip (5 mm). The most important advantage is that it provides ease of use and dissection at the deep pelvis and up to the renal vein, especially in overweight endometrial cancer patients. In addition to being more ergonomic for the surgeon, it will reduce the possibility of damaging vital urinary and gastrointestinal organs during dissection due to its limited lateral thermal spread (4mm). The device’s rapid vessel closure, coagulation, and transection features ensure hemostasis and securely sealed lymphatic vessels [23]. Since the lymphatic vessel wall does not contain smooth muscle and there are not enough coagulation factors in the lymph fluid, it can be considered that sealing the lymphatic vessels together with the surrounding connective tissue may be more effective. The advantage of its ergonomics and long jaw can provide this option during dissection. Lymph vessels in four main localizations are thought to contribute more to lymphocele formation, namely distal of the external iliac artery, distal and proximal of the obturator fossa and at the level of the renal vein (Figures 1–4), sealed at least twice in our cases. Besides, dissection was achieved during the removal of all lymph packages. This technique might reduce the development of symptomatic PL. With the routine use of sentinel lymph node applications all over the world, pelvic lymphocele rates are expected to decrease significantly [24].

Due to the differences in tumor biology and the different nature of the surgeries, we evaluated only endometrial cancers, unlike publications in the literature. They mainly cover three types of gynecological cancers (cervix, uterus, and ovary). For the same reason, sarcomas were also excluded from our study and an evaluation was made in a homogeneous patient group.

The limitations of our study are its retrospective design and the lack of a control group. On the other hand, the homogeneity of the study group regarding histology, surgical technique, and the utilized surgical device is the powerful aspect of this paper.

## 5. Conclusion

We found that using advanced bipolar systems for lymph node dissection in endometrial cancers might be effective and can reduce the formation of PL. This decrement will probably lead to better outcome regarding postoperative period, but long-term objective data is required to prove this hypothesis.

## Figures and Tables

**Table 1:** Demographic and clinical characteristics of the patients.

Characteristic	N = 103

Age (years)	58.6 ± 10.2

BMI (kg/m^2^)	28.1 ± 5.6

Previous abdominal surgery	18 (17.5%)

Operation time (min)	228.0 ± 63

Preoperative Hb (g/dL)	12.4 ± 1.7

Postoperative Hb (g/dL)	11.7 ± 1.5

Drain catheter positive	82 (79.6%)

Endometrial Cancer Stage (FIGO)	
I	69 (67%)
II	8 (7.8%)
III	18 (17.5%)
IV	8 (7.8%)

Adjuvant treatment	
KT	43 (41.7%)
RT	57 (55.3%)

Note: Data are presented as mean ± standard deviation and number (%)

BMI: Body mass index, FIGO: International Federation of Gynecology and Obstetrics, CT: Chemotherapy, RT: Radiotherapy

Hb = hemoglobin

**Table 2:** Tumor characteristics of 103 EC cases.

Endometrial cancer histology	
Endometrioid	87 (84.5%)
Serous	10 (9.7%)
Clear cell	6 (5.8%)

Grade	
I	26 (25.2%)
II	46 (44.7%)
III	31 (30.1%)

Largest tumor diameter	
≤4 cm	61 (59.2%)
>4 cm	42 (40.8%)

LVSI	
Positive	32 (31.1%)
Negative	71 (68.9%)

Localization	
Upper segment	76 (73.8%)
Lower segment	27 (26.2%)

Dissected lymph node number	
Total	36 (9–133)*
Pelvic	24.4 ± 12.2
Paraaortic	14.4 ± 10.9

Metastatic lymph node number	
Total	2 (1–27)*
Pelvic	2 (1–8)*
Paraaortic	2 (1–19)*

Note: Data are presented as mean ± standard deviation and number (%).

LVSI: Lymphovascular space invasion.

* As the distribution is not homogeneous, median (min–max) values are given.

**Table 3:** Comparison of clinicopathologic factors between pelvic lymphocele present and absent cases.

	Lymphocele (+) (n = 24)	Lymphocele (−) (n = 79)	*p-*value
BMI (kg/m^2^)	30.2 ± 6.46	27.55 ± 5.3	***0.045*** *

Histology			
Endometrioid	15 (%17)	72 (%83)	***0.001***
Serous	4 (%40)	6 (%60)	
Clear	5 (%83)	1 (%17)	

Grade			
I	0	26 (%100)	***0.001***
II	11 (%24)	35 (%76)	
III	13 (%42)	18 (%58)	

Largest tumor diameter			
≤4 cm	13 (%21)	48 (%79)	0.565
>4 cm	11 (%26)	31 (%74)	

LVSI			
Positive	12 (%38)	20 (%63)	***0.022***
Negative	12 (%17)	59 (%83)	

Tumor localization			
Upper segment	15(%20)	61 (%80)	0.151
Lower segment	9 (%33)	18 (%67)	

FIGO stage			
I–II	13 (%17)	64 (%83)	***0.008***
III–IV	11 (%42)	15 (%58)	

Adjuvant RT	20 (%35)	37 (%65)	***0.002***

Adjuvant KT	15 (%35)	25 (%65)	***0.019***

Drain catheter			
Positive	21 (%26)	61 (%74)	0.273
Negative	3 (%14)	18 (%86)	

Previous abdominal surgery			
Yes	9 (%50)	9 (%50)	***0.003***
No	15 (%18)	70 (%82)	

Extracted lymph node			
Total number	47 (13–77)	33 (9–133)	***0.005*** **
Pelvic	27.2 ± 9.9	23.53 ± 12.79	*0.194* *
Paraaortic	19.9 ± 10.1	12.85 ± 10.76	***0.008*** *

Metastatic lymph node			
Positive	8 (% 47)	9 (%53)	***0.024***
Negative	16 (%18.6)	70 (%81.4)	

Note: Data are presented as mean ± standard deviation and number (%)

BMI: Body mass index, LVSI: Lymphovascular space invasion, FIGO: International Federation of Gynecology and Obstetrics, RT: Radiotherapy, CT: Chemotherapy,

* Student’s *t*-test was applied because of the normal distribution and the mean standard deviation values were given.

** Because the normal distribution was not achieved, the Mann–Whitney U test was applied and the median (min–max) values were given.
